# Anisotropy visualisation from X-ray diffraction of biological apatite in mixed phase calcified tissue samples

**DOI:** 10.1038/s41598-025-88940-2

**Published:** 2025-02-14

**Authors:** Robert Scott, Iain D. Lyburn, Eleanor Cornford, Pascaline Bouzy, Nicholas Stone, Charlene Greenwood, Sarah Gosling, Emily L. Arnold, Ihsanne Bouybayoune, Sarah E. Pinder, Keith Rogers

**Affiliations:** 1 Cranfield Forensic Institute, Cranfield University, Bedford, UK; 2 Gloucestershire Hospitals NHS Foundation Trust, Cheltenham, UK; 3 Medical Imaging Centre, Cobalt Medical Charity, Cheltenham, UK; 4 School of Physics and Astronomy, University of Exeter, Exeter, UK; 5 School of Chemical and Physical Sciences, Keele University, Keele, UK; 6 Diamond Light Source Ltd., Didcot, UK; 7 School of Cancer and Pharmaceutical Sciences, King’s College, London, UK

**Keywords:** X-ray diffraction, Apatite, Hydroxyapatite, Whitlockite, Anisotropy, Characterization and analytical techniques, Diagnostic markers, Bioinspired materials, Biomineralization

## Abstract

X-ray diffraction is widely used to characterise the mineral component of calcified tissue. Broadening of the diffraction peaks yields valuable information on the size of coherently diffracting domains, sometimes loosely described as crystallite size or crystallinity. These domains are markedly anisotropic, hence a single number describing their size is misleading. We present a novel variation on a method for visualising crystallographic anisotropy in X-ray diffraction data. This provides an intuitively interpretable depiction of crystalline domain size and anisotropy. The new method involves creating a polar plot of calculated domain thickness for peaks in a diffractogram versus crystallographic direction. Points with the least error are emphasised. Anisotropic domain dimensions are calculated by refining an ellipsoidal model in a whole pattern fit. These dimensions are then used to overlay an ellipse on the peak broadening plot. This is illustrated by application of the method to calcifications in breast tissue with suspected cancer, which frequently contain whitlockite as well as nanocrystalline apatite. Like most biogenic apatite, this exhibits markedly anisotropic peak broadening. The nature of this anisotropy offers potentially useful information on normal function and pathology of calcified tissue and is a frequently neglected crystallographic feature of these materials.

**Supplementary Information:**

The online version contains supplementary material available at 10.1038/s41598-025-88940-2.

## Introduction

The mineral phase of calcified tissues is predominantly composed of nanocrystalline calcium phosphate with an apatite structure. The crystallographic parameters of biological apatites have been extensively studied by X-ray diffraction. These parameters are central to understanding the processes of formation and maturation of both normal calcified tissue and of pathological calcifications. There is also accumulating evidence that the crystallographic characteristics of microcalcifications associated with tumours could be of potential diagnostic and prognostic significance.

The crystallographic lattice parameters of apatites are dependent on ionic substitutions within the apatite structure, which is notably able to accommodate a wide range of biologically relevant ions^1^. In particular, levels of carbonate substitution have long been related to pathology^2^, and magnesium substitution is of more recent interest^3,4^. Determination of lattice parameters from diffraction peak positions is comparatively straightforward by conventional pattern fitting methods.

The broadening of peaks in a diffractogram can also give valuable information concerning the nature of the apatite phase. In general, peak broadening can result from a wide range of crystal lattice imperfections, including the finite size of coherently diffracting crystalline domains, and spread in lattice parameter values within the gauge volume, variously termed inhomogeneous lattice strain or microstrain. Peak broadening is challenging to quantify in biological apatites due to extensive overlapping of peaks. Moreover, these measurements can be difficult to interpret since they exhibit strong anisotropy; *hk0* reflections are markedly broader than *00l* reflections^5,6^. Thus, not only is a single value for size and/or strain inadequate to describe the material, but the anisotropy is itself of interest as a material characteristic. In addition, pathological calcifications frequently contain whitlockite in addition to apatite^3^. Overlapping whitlockite and apatite peaks further confound reliable broadening measurements of any given apatite peaks.

Despite the commonly observed crystallographic anisotropy in biological apatites, there have been few attempts to quantify this. Some previous analyses have involved measuring the full width at half maximum (FWHM) of just two distinct peaks: *002* (diffraction vector parallel to the crystallographic c-axis) and *310* (diffraction vector perpendicular to the c-axis)^6–9^. Another analysis used four peaks with a diffraction vector perpendicular or parallel to the c-axis, namely *002*,* 210*,* 300*, and *004*^10^. However, such analyses do not make full use of the data, since this peak selection only uses 2 out of the top 10 highest intensity peaks for apatite.

Many powder diffraction pattern fitting software packages contain some form of anisotropy size or strain fitting routines which can be applied to a whole pattern fit. These may be based, for example, on spherical harmonic expansion^11^ or the Stephen’s model for anisotropic strain broadening^12^. These phenomenological models are primarily used to improve the pattern fit in Rietveld analysis to obtain more reliable structure parameters, rather than as a measure of anisotropic size or strain broadening. These methods have rarely been used for the analysis of biological apatite, and it is difficult to assign a physical meaning to the calculated anisotropy parameters.

There is a long history of applying analytical methods for modelling size broadening as a function of crystallographic direction for a wide range of shapes^13^, such as prismatic and cylindrical crystallites^14^. Calculation of broadening for an ellipsoid is much simpler, since this is a simple linear transform of a sphere^15^, for which the Scherrer constant is the same in all directions. The volume weighted average thickness of an ellipsoid is always ¾ of the diameter in the same direction^16^. For a sphere, and hence an ellipsoid, the Scherrer factor in terms of diameter (for measurements of integral broadening) is 4/3 ^13,17^. In terms of FWHM, a value of 1.107 has been calculated^17^, though of course this does depend on peak shape. Unless crystallite shape is known a-priori, it has been suggested that it is safest to assume that the particle is ellipsoidal^17^. Reverting to fitting a much simpler ellipsoidal shape for the coherently diffracting domain volume has therefore received more recent attention as a technique applied to nanocrystalline materials^16,18,19^.

A simple polar-plot depiction of broadening as a function of crystallographic direction was first proposed by Langford et al.^20^. The method proposed herein builds on this, with the aim of providing a new, intuitively interpretable visualisation of crystalline anisotropy based on a whole pattern fit.

## Materials and methods

### Specimens

This analytical development was undertaken as a component of a study examining ectopic calcifications associated with breast tissues, but the methods could equally well be applied to other biogenic apatites.

Histological breast tissue sections were cut from pseudonymised formalin fixed paraffin embedded blocks selected from the Gloucestershire Hospitals NHS Foundation Trust diagnostic archive and King’s Health Partners Cancer Biobank. These contained image guided biopsy tissue taken to assess suspicious or indeterminate microcalcification in patients recalled following screening mammography. Specimen use was covered by National Health Service HRA/HCRW Ethics Approval (REC reference 20/NW/0057).

Sections were mounted on polyolefin film as previously described^10^. A sample of NIST Standard Reference Material 2910b hydroxyapatite was mounted in a similar way for comparison. Diffraction data was collected on beamline i18 at the Diamond Light Source, Didcot, UK, with a 10 × 10 μm spot size and an energy of 10.0 keV. The scattered photons were recorded using a flat panel Excalibur detector consisting of an 8 × 7 tiled array of Medipix 256 × 256 pixel panes. The detector was calibrated using NIST Standard Reference Material 640c silicon powder.

As illustrative examples of our new approach, diffractograms from two calcifications were selected with widely differing values of peak broadening. Calcification A was from a specimen with a pathology opinion code of B5a – Ductal Carcinoma in-situ. Calcification B was from a specimen with a pathology opinion code of B2 – Benign. In addition, a diffractogram from the NIST standard hydroxyapatite was included for reference. As another illustration, calcifications representing the extreme values of domain aspect ratio were selected; Calcification C was from a specimen with B5b – Invasive Carcinoma, and Calcification D was from a specimen with a pathology opinion code of B2 – Benign.

### Analysis

Diffraction images were reduced to 1D diffractograms using the DAWN software suite^21^. Pattern fitting was subsequently conducted in TOPAS 6.0 ^22^. All charts were created in R (v 4.4.0)^23^. The silicon standard material was used to measure the magnitude of instrumental broadening, and the 2θ dependence was refined in a Pawley fit of the NIST hydroxyapatite standard. The calculated instrumental broadening increased from 0.047° to 0.087° over a 2θ range of 19° to 44°. FWHM correction used the Thompson approximation for the convolution of Gaussian and Lorentzian functions^24^.

An initial analysis consisted of a whole pattern Rietveld fit for hydroxyapatite and whitlockite phases over a 2θ range of 19° to 44° (d-spacing 3.756 to 1.655 Å). This gave an estimate of the weight% of whitlockite present and the lattice parameters for both the apatite and whitlockite phases. A Stephens phenomenological model for peak broadening^12^ was applied to the apatite phase; this was solely to improve the whole pattern fit in the presence of anisotropic broadening, and hence gave a more precise initial estimate of lattice parameters. The output of this phase consisted of all the fitted whitlockite parameters and the apatite lattice parameters.

Further analyses were conducted using the initial estimate of the apatite lattice parameters from the first stage. The whitlockite phase refined in the initial stage was included with fixed parameters and no further refinement. This is equivalent to subtracting the whitlockite from the pattern.

To measure peak broadening as a function of crystallographic direction, the apatite phase was fitted with all 24 peaks listed in the NIST Standard Reference Material 2910b (Hydroxyapatite) certificate of analysis, in the range *002* to *004*. The peak positions were calculated from the apatite lattice parameters, which were further refined from a starting value taken from the preliminary phase analysis. Peaks were fitted with a pseudo-Voigt profile, individually refined for intensity, width and Lorentzian mixing factor. In this way, the peak shapes were independent, but positions constrained by the refined lattice parameters.

An additional analysis was conducted in which the Lorentzian mixing factor was refined to a single value in the range 0 to 1 for the whole pattern, rather than independently for each peak. Results were used to determine the extent to which peak broadening was Lorentzian or Gaussian in nature in these materials.

The output included individual peak FWHM values. The apparent coherent domain size parallel to the diffraction vector for each peak was calculated from the Scherrer equation:

1 $$\tau _{{hkl}} = \frac{{K \cdot \lambda }}{{H_{{hkl}} \cdot \cos \theta }}$$

τ_*hkl*_ = calculated coherent domain thickness perpendicular to plane *hkl*. H_*hkl*_ = measured FHWM for *hkl* peak, corrected for instrumental broadening. K = Scherrer shape factor = 1.1 for thickness of ellipsoid and FWHM^17,25^. λ = X-ray wavelength (1.2398 Å).

For hexagonal materials such as hydroxyapatite, the angle between the diffraction vector for each *hkl* plane and the crystal ‘c’ axis can be calculated as follows:

2 $$\cos \left( {\varphi _{{hkl}} } \right) = l \cdot \left( {\frac{{d_{{hkl}} }}{c}} \right)$$

3 $$\frac{1}{{d_{{hkl}} }} = \sqrt {\frac{{4\left( {h^{2} + hk + k^{2} } \right)}}{{3a^{2} }} + \frac{{l^{2} }}{{c^{2} }}}$$

where a, c = lattice parameters. d_*hkl*_ = interplanar spacing for *hkl*. φ_*hkl*_ = angle between normal to *hkl* plane and the ‘c’ axis.

The calculated domain sizes corresponding to these 24 peaks were then plotted on a polar plot each with radius = 0.5 x τ_*hkl*_ and angle from the ordinate = φ_*hkl*_. In order to create a visual representation of the domain shape and size, the points were repeated in all four quadrants of the polar plot. To emphasise the points with least error, the area of the plotted points was set to be inversely proportional to the reported error in FWHM^26^.

In the Langford representation^20^, a least-squares curve was fitted through the points, with a form calculated from the relationship between angle and volume-weighted thickness for a given crystallite shape model (e.g. cylindrical). Our proposed method takes a different approach to creating a fitted line. For this, the 24 selected hydroxyapatite peaks are fitted in a least-squares whole-pattern fit, but with the FWHM values constrained by an ellipsoidal model for anisotropic domain size, with the refined ‘ab’ and ‘c’ domain size as outputs. The domain thickness for each peak was constrained using the polar form for an ellipse:

4 $$\tau _{{hkl}} = \frac{{\tau _{a} \cdot \tau _{c} }}{{\sqrt {(\tau _{a} \cdot \cos \left( {\varphi _{{hkl}} } \right))^{2} + (\tau _{c} \cdot \sin \left( {\varphi _{{hkl}} } \right))^{2} } }}$$

where τ_*hkl*_ = apparent coherent domain thickness perpendicular to plane *hkl*. τa, τc = refined coherent domain size parallel to ‘a’ and ‘c’ axes. φ_*hkl*_ = angle between plane *hkl* and the ‘c’ axis

The FWHM of each peak was calculated from the ellipsoidally constrained apparent coherent domain thickness, and used in the pattern fit. The fit was refined by varying the domain thickness parallel and perpendicular to the ‘c’ axis. As with the previous analysis, the Lorentzian mixing factor of the pseudo-Voigt peaks was refined individually for each peak, and lattice parameters were also refined from the initial fit values. This differs from the Langford approach in that the plotted ellipse is not a least-squares fit to the individual peak domain size, but least-squares whole pattern Pawley fit. Thus, the overlay of calculated domain thickness for individually fitted peaks gives simple graphical check of the calculated anisotropy parameters calculated from the whole pattern.

## Results

**Fig. 1:**
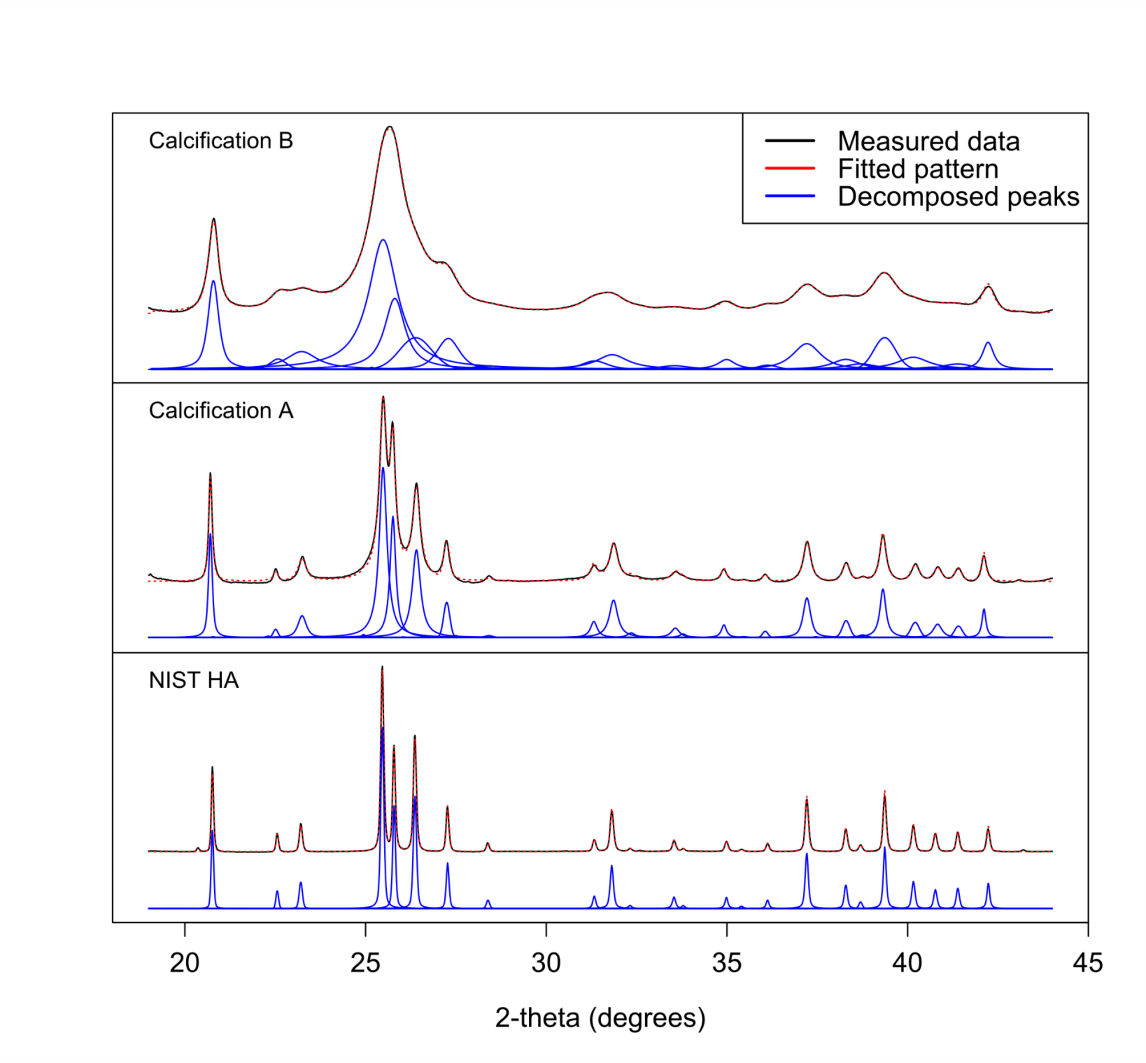
Diffractograms after background subtraction, showing the measured intensity in black, and the fitted pattern as a dashed red line. This is composed of 24 fitted apatite peaks individually refined for intensity, width and Lorentzian mixing factor, shown in blue.

Diffractograms from three selected specimens are shown in Fig. 1 including two typical diffractograms from breast tissue calcifications. These exhibit peak broadening towards the broad and narrow extremes of the range observed during the study consisting of > 4000 diffractograms. A diffractogram from the NIST hydroxyapatite standard is also shown for reference. The plots show the measured intensity after background subtraction, with the fitted pattern overlaid. The decomposed apatite peaks are shown vertically offset for clarity. It is clearly evident that the sum of these decomposed peaks gives a good fit of the refined pattern to the measured data.

**Fig. 2:**
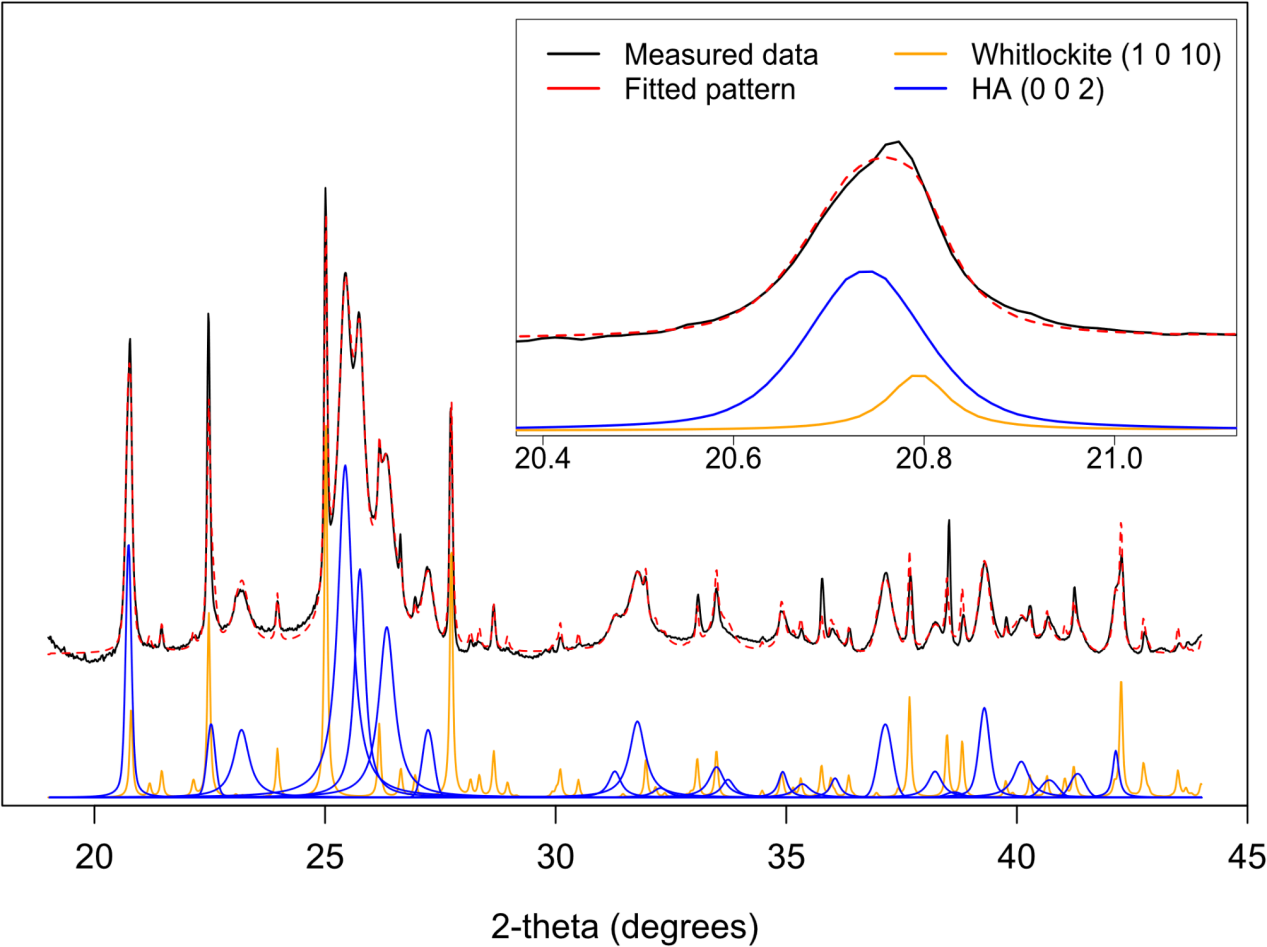
Diffractogram of a calcification containing 20 wt% whitlockite. The inset view of the 002 apatite peak and the *1 0 10* whitlockite peak shows the potential for confounding if the whitlockite peak is not included in the fit.

A diffractogram from a calcification containing ~ 20 wt% whitlockite is shown in Fig. 2, with an expanded view of the hydroxyapatite 002 peak. Unless whitlockite is adequately modelled and included in the pattern fit, both position and broadening HA peaks will be erroneous, particularly for the influential HA *002*, which overlaps with the whitlockite *1 0 10* peak. This underlines the importance of the first stage fit of HA and whitlockite in the case of pathological calcifications, in which whitlockite is frequently present^3^.

The pattern fit in which peak shape was modelled using a single refined value of Lorentzian mixing factor showed that broadening in these calcifications was overwhelmingly Lorentzian in nature. Calcifications A-D had a mean Lorentzian mixing factor of 0.97 (range 0.91–1.00). There is both a theoretical justification and experimental evidence that the Lorentzian (Cauchy) component of the peak profiles is due to size effects whereas the Gaussian contribution arises from microstrain^27^. It is therefore reasonable to assume that in these nanocrystalline materials, size broadening dominates over strain broadening. Further analysis was therefore conducted in terms of size broadening only. In contrast, the single refined Lorentzian mixing factor for the NIST hydroxyapatite specimen was 0.57 (Std.Dev. 0.007). From application of Eq. 31 in^28^, the Lorentzian broadening represents approximately 70% of the total broadening, hence the calculated domain size underestimates the true domain size by about 30%. This synthetic standard material is not the main subject of this investigation, hence further analysis of anisotropic size and strain was not performed.

**Fig. 3:**
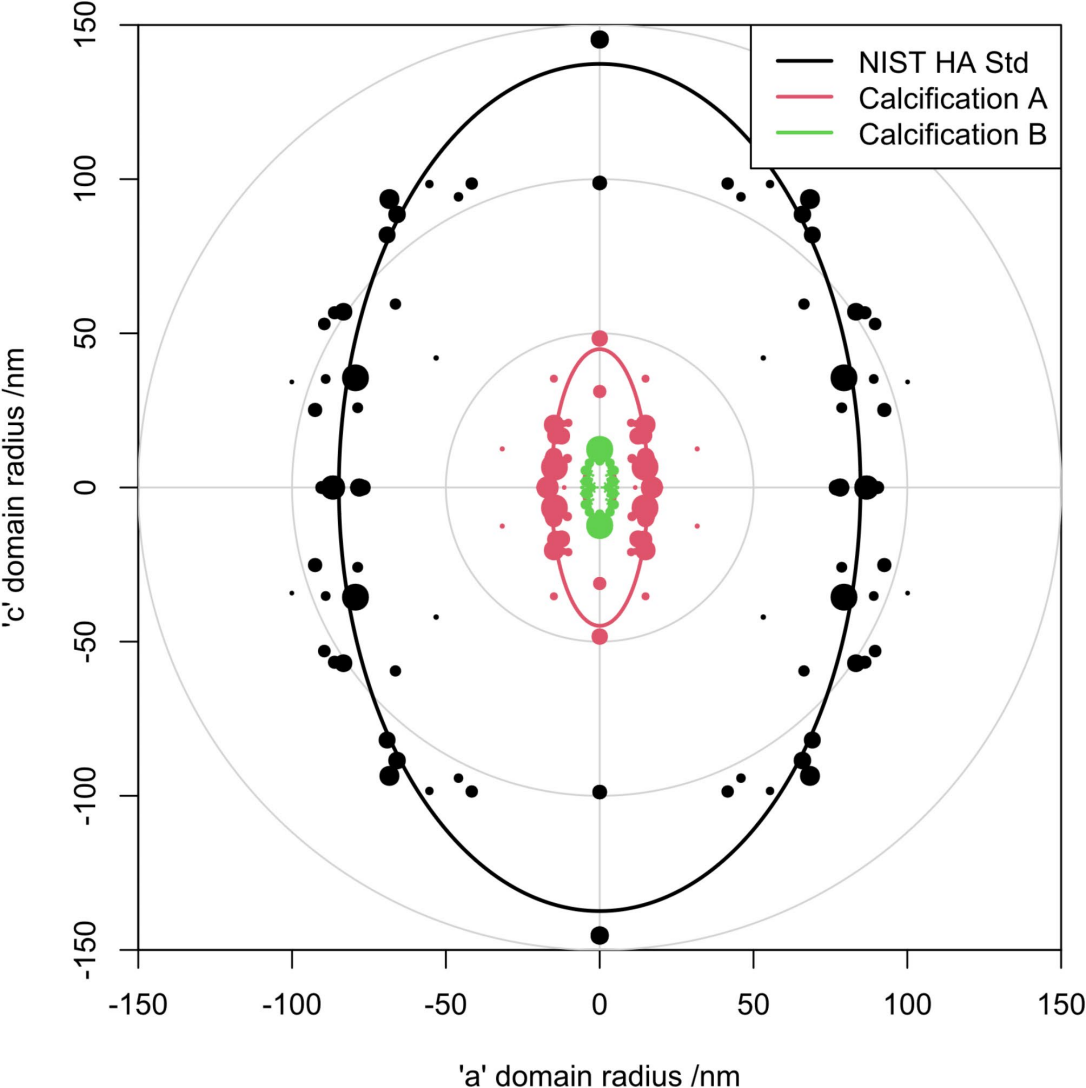
Polar plot showing calculated domain thickness for each of 24 apatite peaks as a function of crystallographic direction, overlaid with an ellipse representing the domain dimensions calculated from an ellipsoidal whole pattern fit. The diameter of the plotted points is inversely proportional to the calculated error in peak width measurement. FWHM values used to calculate both the point positions and the ellipsoidal fit were corrected for instrumental broadening.

The measured individual peak FWHM values were used to calculate domain thickness in the crystallographic direction corresponding to each peak. The dimensions are represented on a polar plot shown in Fig. 3 for the same three diffractograms presented in Fig. 1.

The ordinate on the plot represents the crystallographic c-axis. The data has been reflected into each quadrant to give a direct visual representation of the calculated dimensions of the coherently diffracting domain. Note that the peak FWHM values used to calculate the position of these points are unconstrained by the ellipsoidal model. There was a large variation in relative error of the FWHM values, and this is reflected by the variation in area of the plotted points. The plotted ellipses are derived from whole-pattern least-squares fits in which the FWHM of each peak is constrained by the ellipsoidal model. Calculated domain dimensions for each of these specimens is shown in Table 1.

**Table 1:** Coherently diffracting apatite domain dimensions from the whole pattern ellipsoidal fit for the calcifications illustrated in Figs. 3 and 4.

Specimen	Domain size/nm		Aspect ratio
	ab-plane	c-axis	
Calcification A	31.5	89.7	2.9
Calcification B	7.6	23.4	3.1
Calcification C	8.0	31.5	3.9
Calcification D	7.9	16.2	2.0
NIST 2910b Hydroxyapatite	169.6	274.8	1.6

**Fig. 4:**
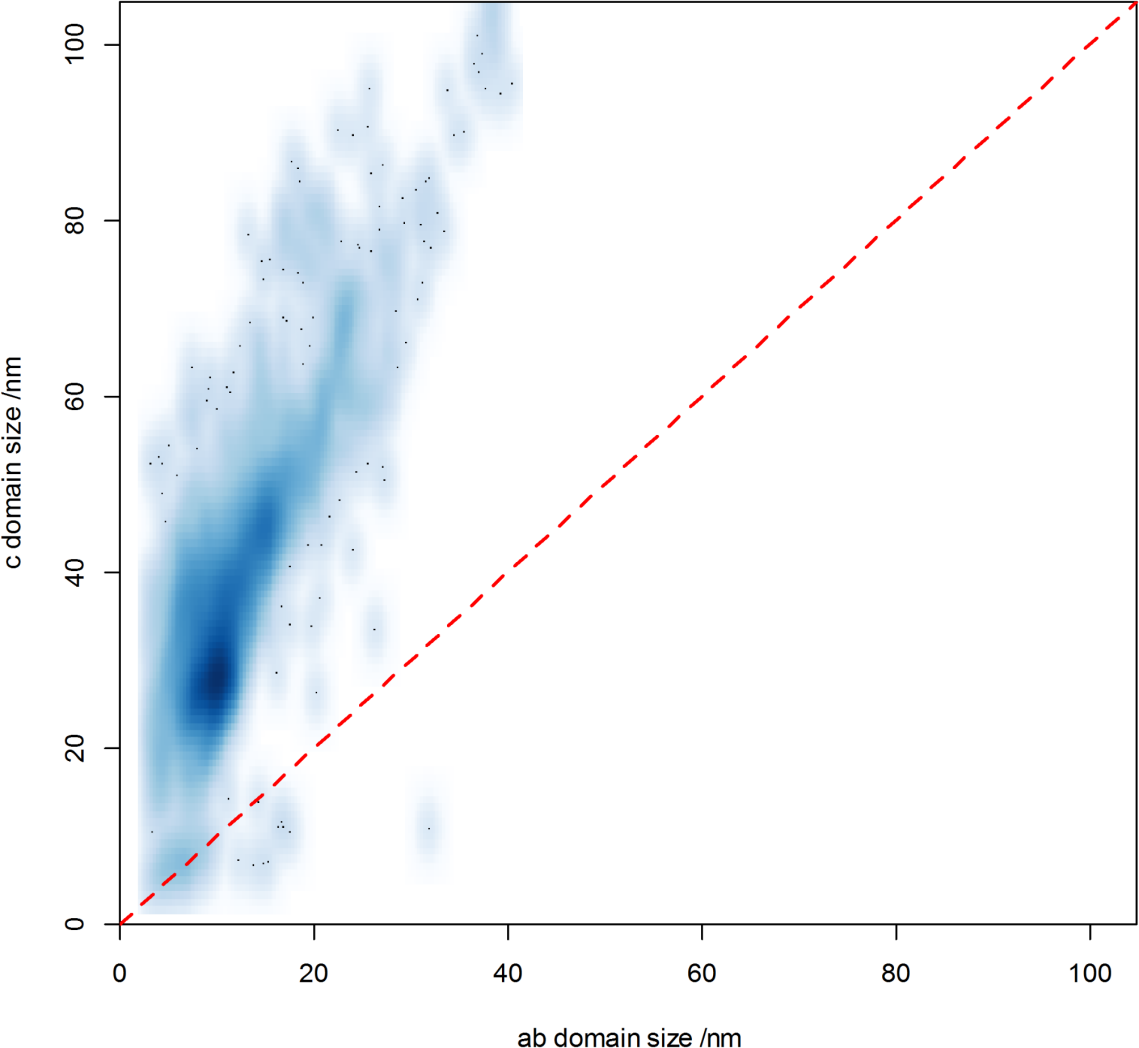
2D kernel-density plot of ellipsoidal domain dimensions for 4125 diffractograms measured from taken from 259 calcifications.

Polar plots of additional specimens can be seen in supplementary material, Fig. S1. As further corroboration of the method, thickness values derived from the ellipsoidally constrained fitted FHWM value for each peak were also plotted. These all lie exactly along the locus of ellipses constructed from the refined major and minor domain dimensions. An example can be seen in supplementary material, Fig. S2.

**Fig. 5:**
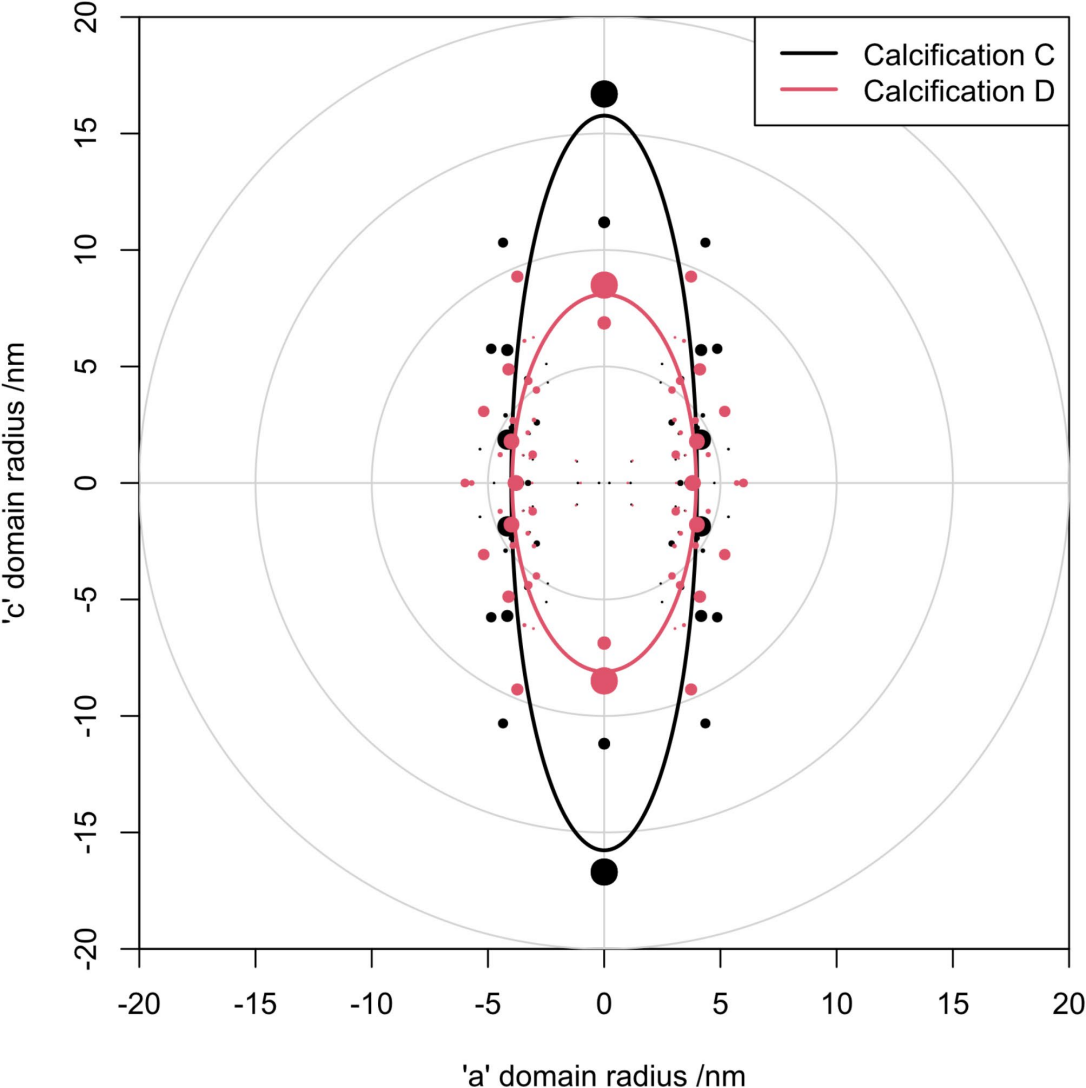
Polar plot similar to Fig. 3, showing the fit for calcifications close to the extremes of aspect ratio for these specimens.

In these biogenically derived materials, there is substantial variation in calculated aspect ratio of the domains. This is illustrated in Fig. 5, and polar plots of diffractograms with a high and low degree of anisotropy are shown in Fig. 4.

## Discussion

It is clear from examination of the diffractograms in Fig. 1 that the NIST hydroxyapatite standard material exhibits the least broadening and Calcification B the most. Decomposition of the overlapping peaks reveals that the broadening within each diffractogram varies between peaks, particularly for Calcifications A and B. However, it is not self-evident from the diffractograms whether peak broadening varies systematically with crystallographic direction, and if so, what form this anisotropy takes.

In isotropic materials, a Williamson Hall plot or one of its derivatives^29^ is a useful tool for quantifying both size and microstrain based on peak broadening measurements. Anisotropy makes this problematic^30^. In an isotropic material, the points should lie along a line. Microstrain can be calculated from the gradient of the points, and domain size from the intercept. Anisotropy results in a vertical spread in points as a function of crystallographic direction. Grouping points by crystallographic direction can give some indication of the size and strain contributions to broadening in different directions, but interpretation is far from easy or intuitive, and can only be applied to a subset of peaks, which are often few in number. Errors of peak broadening measurement propagate to create additional scatter and further complicates the interpretation. A cloud of points rather than a line does not constitute evidence of anisotropy per se, since it could be mostly or entirely a result of peak broadening measurement errors.

An alternative approach would be to include both anisotropic size and anisotropic microstrain parameters in refinement of a whole pattern fit. In practice, separating anisotropic microstrain from anisotropic domain size proved impossible in this data set given the broad overlapping peaks in nanocrystalline biological apatite, and the limited diffraction angle measurable with a flat panel detector.

In cases such as this where anisotropy complicates separation of size and strain effects, single-line peak-shape methods can be useful^31^. The Lorentzian nature of broadening observed in these nanocrystalline biological apatite specimens indicates that size effects dominate.

The plot in Fig. 3 clearly shows that, as well as having a smaller apparent domain size, the aspect ratio of the calcifications is substantially larger than that of the NIST hydroxyapatite standard. The consistency of the ellipses from the constrained fit and the points from the unconstrained fit give a measure of confidence in the calculated domain size and aspect ratio. In recent years there has been a shift from reporting simply mean and standard error towards plotting all the supporting data points^32,33^. This proposed method for plotting size anisotropy in X-ray diffraction fits with this trend in that the individual peak broadening measurements are plotted along with the calculated anisotropy from a whole pattern fit.

The physical shape of apatite crystallites in calcified tissue is controversial^34,35^, but it is generally agreed that the crystallites are rarely equiaxed. The aspect ratio of these may be of biological significance. For instance, direct observation of the shape and size of precipitated carbonated apatite crystallites shows that carbonate limits the size of the crystals and makes them grow more equiaxed than needle-like^36^. In addition, anisotropic crystallite morphology is an important characteristic in biomimetic biomaterials^37^.

This method could readily be extended to data acquired from X-ray diffraction computed tomography of bones and teeth^38^. This could be used to construct maps of anisotropy in tomographic sections. There was no evidence of preferred orientation in the specimens in this study. However, bones and teeth can exhibit marked preferred orientation^38,39^. The use of a 2D panel detector would enable pole figures to be constructed, and texture coefficients correlated with the measured anisotropy.

In summary, fitting of an ellipsoidal model of size broadening in X-ray diffraction studies of biological apatite gives a quantitative measure of anisotropy. The proposed method for plotting anisotropy creates an intuitively interpretable depiction of coherently diffracting domain size and aspect ratio. These parameters provide potentially useful information on normal function and pathology in calcified tissue, and of disease processes in soft tissue calcifications.

## Electronic supplementary material

Below is the link to the electronic supplementary material.

Supplementary Material 1

## Data Availability

Data supporting this study are openly available from the Cranfield University CORD database at DOI https://doi.org/10.57996/cran.ceres-2623. This study did not involve interaction with any human subjects.
