# Supplementary material for: Anisotropy visualisation from X-ray diffraction of biological apatite in mixed phase calcified tissue samples
Source: Sci Rep. 2025 Feb 14;15:5478. doi: 10.1038/s41598-025-88940-2 (PMC11828961; doi:10.1038/s41598-025-88940-2)
Supplement: Supplementary file 1 — Supplementary Material 1 [file 41598_2025_88940_MOESM1_ESM.pdf]

Anisotropy visualisation from X-ray diffraction of biological apatite in mixed phase samples

Supplementary Material

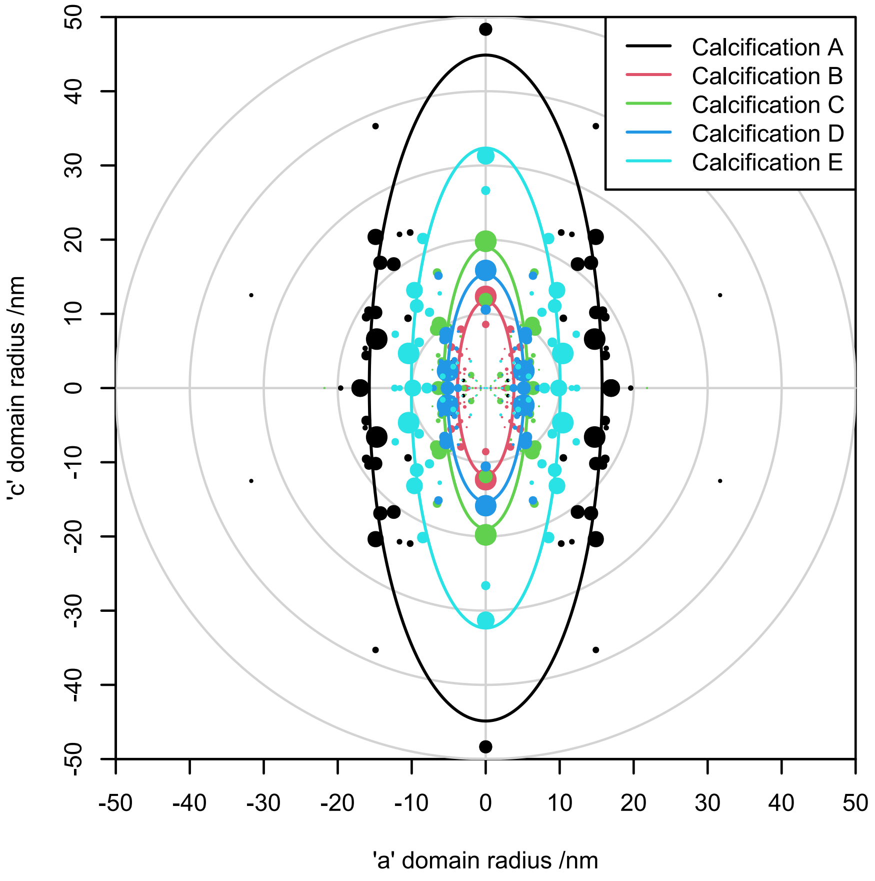

Supplementary Figure S1: Additional polar plot from further specimens of showing calculated domain thickness for each of 24 apatite peaks as a function of crystallographic direction, overlaid with an ellipse representing the domain dimensions calculated from an ellipsoidal whole pattern fit. The diameter of the plotted points is inversely proportional to the calculated error in peak width measurement.

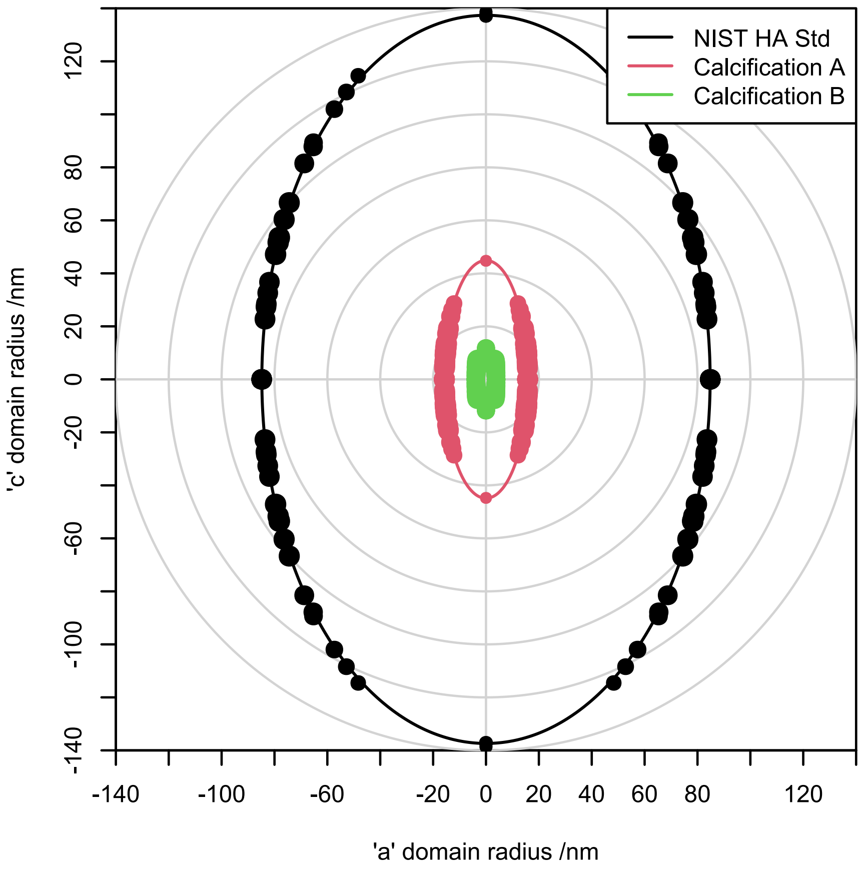

Supplementary Figure S2 Polar plot of individual peaks in the ellipsoidally constrained model, confirming that these lie along the perimeter of the ellipse with refined dimensions from the constrained model.
